# Mitochondrial genome and nuclear ribosomal RNA analysis place *Alveonasus lahorensis* within the Argasinae and suggest that the genus *Alveonasus* is paraphyletic

**DOI:** 10.1017/S0031182024000441

**Published:** 2024-08

**Authors:** Ben J. Mans, Lidia Chitimia-Dobler, Ronel Pienaar, Minique de Castro, Mehran Khan, Mashal M. Almutairi, Abdulaziz Alouffi, Abid Ali

**Affiliations:** 1 Epidemiology, Parasites and Vectors, Agricultural Research Council-Onderstepoort Veterinary Research, Onderstepoort 0110, South Africa; 2 Department of Life and Consumer Sciences, University of South Africa, Florida 1709, South Africa; 3 Department of Zoology and Entomology, University of the Free State, Bloemfontein 9301, South Africa; 4 Department of Virology and Rickettsiology, Bundeswehr Institute of Microbiology, Munich, Germany; 5 Department of Infection and Pandemic Research, Fraunhofer Institute of Immunology, Infection and Pandemic Research, Penzberg, Germany; 6 The Biotechnology Platform, Agricultural Research Council-Biotechnology Platform, Onderstepoort 0110, South Africa; 7 Department of Zoology, Abdul Wali Khan University Mardan, Mardan 23200, Pakistan; 8 Department of Pharmacology and Toxicology, College of Pharmacy, King Saud University, Riyadh 11451, Saudi Arabia; 9 King Abdulaziz City for Science and Technology, Riyadh 12354, Saudi Arabia

**Keywords:** Argasidae, Argasinae, *Alveonasus lahorensis*, molecular systematics, Ornithodorinae

## Abstract

Two major families exist in ticks, the Argasidae and Ixodidae. The Argasidae comprise 2 sub-families, Argasinae and Ornithodorinae. The placement into subfamilies illuminate differences in morphological and molecular systematics and is important since it provides insight into evolutionary divergence within this family. It also identifies fundamental gaps in our understanding of argasid evolution that provide directions for future research. Molecular systematics based on mitochondrial genomics and 18S/28S ribosomal RNA confirmed the placement of various genera and subgenera into the Argasinae: *Argas* (including *Argas* and *Persicargas*), *Navis*, *Ogadenus*, *Otobius lagophilus*, *Proknekalia*, *Secretargas* and the Ornithodorinae: *Alectorobius*, *Antricola* (including *Antricola* and *Parantricola*), *Carios*, *Chiropterargas*, *Nothoaspis*, *Ornithodoros* (including *Microargas*, *Ornamentum*, *Ornithodoros* sensu strictu, *Pavlovskyella*), *Otobius* sensu strictu, *Reticulinasus* and *Subparmatus*. The position of *Alveonasus* remains controversial since traditional taxonomy placed it in the Ornithodorinae, while cladistic and limited molecular analysis placed it in the Argasinae. The current study aimed to resolve the systematic position of *Alveonasus* using mitochondrial genomic and 18S/28S ribosomal RNA systematics by sequencing the type species *Alveonasus lahorensis* from Pakistan. In addition, the mitochondrial genomes for *Argas reflexus* and *Alectorobius kelleyi* are reported from Germany and the USA, respectively. The systematic data unambiguously place *Alveonasus* in the Argasinae and also suggest that *Alveonasus* may be another paraphyletic genus.

## Graphical abstract

**Figure:**
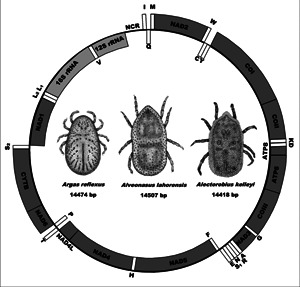

## Introduction

Ticks (Ixodida) are comprised of 3 extant families, Argasidae (soft ticks), Ixodidae (hard ticks) and Nuttalliellidae and 2 extinct families, Deinocrotonidae and Khimairidae (Mans, 2023). The Argasidae is divided into the Argasinae and Ornithodorinae subfamilies (Mans, 2023). The Ornithodorinae currently comprise the genera *Alectorobius*, *Antricola* (including *Parantricola*), *Carios*, *Chiropterargas*, *Nothoaspis*, *Ornithodoros* (including *Microargas*, *Ornamentum*, *Ornithodoros* and *Pavlovskyella*), *Otobius*, *Reticulinasus* and *Subparmatus* (Mans *et al*., 2021; Mans, 2023). The Argasinae currently comprise the genera *Alveonasus*, *Argas* (including *Argas* and *Persicargas*), *Navis*, *Ogadenus*, *Proknekalia* and *Secretargas* (Mans *et al*., 2021; Mans, 2023). *Otobius lagophilus* Cooley and Kohls, 1940 has recently been shown to group in the Argasinae even though *Otobius* sensu stricto group within the Ornithodorinae (Kneubehl *et al*., 2022). Understanding the relationships between the families and the relationships between genera within the families is important for an understanding of tick evolution (Mans *et al*., 2016; Mans, 2023). Generic relationships in the Argasidae in particular is to a large extent unresolved, while many genera seem to be paraphyletic or polyphyletic, leading to the possibility that argasid genera may increase in the future. A number of genera and species have been shown to group outside of their traditional placement in the argasid subfamilies, notably *Proknekalia* and *Otobius lagophilus* in the Argasinae and *Carios* and *Chiropterargas* in the Ornithodorinae (Mans *et al*., 2019, 2021; Kneubehl *et al*., 2022). Placement of argasid genera and species within the subfamilies is therefore important and has not been confirmed for the genera *Alveonasus* or *Microargas*. The genus *Alveonasus* is of particular interest since the major systematic schools have differed regarding its affinity to the various subfamilies (Pospelova-Shtrom, 1969; Keirans *et al*., 1977; Klompen and Oliver, 1993).

The genus *Alveonasus* (Schulze, 1941) is characterized by a non-mammilated body integument with numerous depressions around which wrinkled ridges radiate to give a madreporean sculpturing effect (Clifford *et al*., 1964). The type species is *Alveonasus lahorensis* (Neumann, 1908) (Schulze, 1941). The genus is composed of 8 species and is mainly found in the Afrotropic and Palearctic regions, although *Alveonasus cooleyi* (McIvor, 1941) derives from the Nearctic (Table 1). *Alveonasus lahorensis* is unique in the Argasidae in that it is a 2-host tick species with larvae feeding and detaching as engorged third-instar nymphs that then moult to adults (Hoogstraal, 1985). Hosts include cattle, sheep, camels and wild ungulates. Its geographic distribution covers a wide range of the Palearctic region including Central Asia (southern former USSR), East Asia (China and Tibet), South Asia (Kashmir and Pakistan), Southwest Asia (Iran to Saudi Arabia) and Southeast Europe (Turkey, Bulgaria, Yugoslavia and Greece) (Hoogstraal, 1985).

**Table 1.** Current species placed in the genus *Alveonasus* and their geographic distribution. Specific type localities are also indicated.

Species	Geographic distribution	Inclusion in *Alveonasus*
*Alveonasus acinus* (Whittick, 1938)	Somalia (Type locality: Bulleh Tir)	Pospelova-Shtrom (1953)
*Alveonasus buettikeri* (Vial and Camicas, 2009)	Oman (Type locality: Jabal Qamr)	Vial and Camicas (2009)
*Alveonasus canestrinii* (Birula, 1895)	Iran, Turkmenistan (Type locality: Tehran, Iran)	Grebenyuk (1951)
*Alveonasus cooleyi* (McIvor, 1941)	USA (Type locality: Rox, Nevada)	Pospelova-Shtrom (1953)
*Alveonasus delanoei* (Roubaud and Colas-Belcour, 1931)	Egypt, Morocco (Type locality: Morocco)	Pospelova-Shtrom (1953)
*Alveonasus eboris* (Theiler, 1959)	South Africa (Type locality: Skeerpoort, South Africa)	Theiler (1959)
*Alveonasus foleyi* (Parrot, 1928)	Algeria, Chad, Egypt, Libya, Niger, Morocco, Tunisia (Type locality: Algeria)	Pospelova-Shtrom (1953)
*Alveonasus lahorensis* (Neumann, 1908) TYPE	Palearctic (Type locality: Lahore, Pakistan)	Schulze (1941)

*Alveonasus* was placed within the Ornithodorinae by the Russian (Pospelova-Shtrom, 1946, 1969), American (Clifford *et al*., 1964; Hoogstraal, 1985; Guglielmone *et al*., 2010) and French (Camicas and Morel, 1977; Camicas *et al*., 1998) schools, mostly based on the fact that its body margin is rounded and lack a sutural groove. However, cladistic analysis based on 83 biological and morphological characters placed *Alveonasus* in the Argasinae (Klompen and Oliver, 1993). Phylogenetic analysis of the nuclear 18S ribosomal RNA gene (Black *et al*., 1997; Mans *et al*., 2019, 2021) and the mitochondrial 12S and 16S ribosomal RNA genes (Zhao *et al*., 2018), also placed *Alveonasus* within the Argasinae. Based on these considerations, *Alveonasus* was placed within the Argasinae even though analysis of its full mitochondrial genome was not presented (Mans *et al*., 2019, 2021). The current study sequenced the mitochondrial genome and full-length 18S and 28S ribosomal RNA of *A. lahorensis*, the type species of this genus from Pakistan. The results support the placement of *Alveonasus* within the Argasinae based on both nuclear and mitochondrial gene analysis. In addition, we report the mitochondrial genomes of the type species for *Argas*: the pigeon tick, *Argas reflexus* Fabricius, 1794 from Germany and the bat tick, *Alectorobius kelleyi* (Cooley and Kohls, 1941) from the USA.

## Materials and methods

### Ticks and datasets

The ticks analyzed in the current study include *A. lahorensis* collected from sheep in Khyber Pakhtunkhwa, Pakistan (collected and identified by Abid Ali in 2022) and *A. reflexus,* historical collection and identification by Hans Dautel in Berlin, Germany. In both cases, voucher specimens have been deposited in the Gertrud Theiler National Tick Collection (Agricultural Research Council – Onderstepoort Veterinary Research). The data for *A. kelleyi* was obtained from the NCBI SRA database (SRR23908069) (Occi *et al*., 2023). Tick nomenclature is used according to the proposals by Mans *et al*. (2019) and Mans *et al*. (2021).

### Next-generation sequencing, assembly and mitochondrial genome annotation

Genomic DNA was extracted using the QIAamp DNA Blood Mini Kit (Qiagen), processed using the MGIEasy Universal DNA Library Prep kit (MGI, Shenzhen, China) and sequenced on the MGI DNBSEQ-G400 sequencing instrument using the PE150 (paired-end 2 × 150 bp) format (Agricultural Research Council-Biotechnology Platform, South Africa). Paired-end sequence data were quality trimmed (0.001 quality limit) and MGI adapters were removed using CLC Genomics Workbench v.20.1 software (Qiagen). Standard assembly parameters (mismatch cost-2, insertion cost-3, deletion cost-3, length fraction-0.9, similarity-0.9, minimum contig length-200 and automatic bubble size) were used and assembly was performed using a word size of 49 in CLC Genomics Workbench v.20.1 software (Qiagen). Contigs were identified as mitochondrial, 18S or 28S rRNA using BLASTN analysis (Altschul *et al*., 1990). Final contigs were obtained by mapping data back to the contigs using CLC Genomics Workbench v.20.1 (mismatch cost-2, insertion cost-3, deletion cost-3, length fraction-0.5 and similarity-0.9), to obtain consensus sequences and final coverage values. The mitochondrial genome was annotated using the MITOS and ARWEN servers to identify tRNA genes (Laslett and Canbäck, 2008; Bernt *et al*., 2013). Protein-coding genes were identified using the Expasy Translation Server (https://web.expasy.org/translate/) and BLASTP analysis (Altschul *et al*., 1990).

### 16S rRNA phylogenetic analysis

Sequences of *A. lahorensis*, *A. reflexus* and *A. kelleyi* were used to download the top 100 most closely related sequences from GenBank using BLASTN analysis (Altschul *et al*., 1990). Sequences from the BLASTN analysis were combined to produce a non-redundant dataset with sequences that represent unique species. This yielded a final dataset of 107 sequences that was aligned using MAFFT taking rRNA secondary structure into account (Q-INS-i) (1PAM/k = 2 scoring matrix) (Katoh and Standley, 2013). Maximum likelihood analysis was performed using IQ-Tree2 v 2.2.0 (Minh *et al*., 2020) with an alignment size of 336 bp. The most optimal substitution model used was GTR + F + I + G4. Nodal support was estimated using ultrafast bootstrap (*n* = 10 000) and the 50% consensus tree was reported.

### Cytochrome oxidase I phylogenetic analysis

Sequences of *A. lahorensis*, *A. reflexus* and *A. kelleyi* were used to download the top 100 most closely related sequences from GenBank using BLASTN analysis (Altschul *et al*., 1990). Sequences from the BLASTN analysis were combined to produce a non-redundant dataset with sequences that represented unique species. This yielded a final dataset of 107 sequences that was aligned using MAFFT taking rRNA secondary structure into account (Q-INS-i) (1PAM/*k* = 2 scoring matrix) (Katoh and Standley, 2013). A neighbour-joining analysis was performed in Mega 5 (Tamura *et al*., 2011), using the amino acid matrix derived from the nucleotide sequence with the Jones–Taylor–Thornton amino acid substitution model with uniform rates across sites. Nodal support was estimated using 10 000 bootstraps.

### Mitochondrial genome phylogenetic analysis

Translated protein sequences for the ATP synthase 6 (ATP6), ATP synthase 8 (ATP8), cytochrome oxidase I (COX1), cytochrome oxidase II (COX2), cytochrome oxidase III (COX3), cytochrome b (Cytb), NADH dehydrogenase subunit 1 (ND1), NADH dehydrogenase subunit 2 (ND2), NADH dehydrogenase subunit 3 (ND3), NADH dehydrogenase subunit 4 (ND4), NADH dehydrogenase subunit 4L (ND4L), NADH dehydrogenase subunit 5 (ND5) and NADH dehydrogenase subunit 6 (ND6) genes were used for phylogenetic analysis (Mans *et al*., 2012). Multiple sequence alignments for each protein were performed separately using MAFFT with iterative alignment (FFT-NS-i) and the BLOSUM62 amino acid scoring matrix (Katoh and Standley, 2013). Maximum likelihood analysis was performed in IQ-Tree2 IQ-Tree2 v 2.2.0 (Minh *et al*., 2020). An optimal substitution model was calculated for each protein partition: ATP6 (mtMet + F + R5), ATP8 (mtVer + F + G4), COX1 (mtART + R5), COX2 (mtMAM + F + I + I + R4), COX3 (mtART + F + I + I + R5), CYTB (mtMet + I + G4), NAD1 (mtZOA + F + I + I + R5), NAD2 (mtMAM + F + I + I + R6), NAD3 (mtMet + I + G4), NAD4 (mtMet + F + I + I + R5), NAD4L (mtMet + F + G4), NAD5 (mtMet + F + I + I + R6) and NAD6 (mtVer + F + G4). Absent protein genes were treated as missing data. An edge-proportional partition model with proportional branch lengths (-spp) was used to allow each partition its own specific rate to accommodate different evolutionary rates between partitions. Nodal support was estimated using ultrafast bootstrap (*n* = 1 000 000) and the 50% consensus tree was reported.

### 18S-28S Ribosomal RNA phylogenetic analysis

The 18S and 28S rRNA genes from the Ixodida were downloaded from GenBank and only included in the analysis where both sequences were available for a species. Sequences were cleaned up to include a single representative for each species, except for *A. lahorensis* where the 18S rRNA gene from Black *et al*. (1997) was also included. The 18S and 28S rRNA genes were aligned separately with MAFFT taking rRNA secondary structure into account (Q-INS-i) (1PAM/*k* = 2 scoring matrix) (Katoh and Standley, 2013). GBLOCKS was used to remove columns with less than 50% coverage (Castresana, 2000) resulting in alignments of 1014 bp for the 18S and 540 bp for the 28S rRNA genes. Maximum likelihood analysis was performed using IQ-Tree2 IQ-Tree2 v 2.2.0 (Minh *et al*., 2020). The most optimal substitution model (18S: TIM3e + I + I + R2; 28S: TPM3 + I + I + R2) for each alignment was automatically selected. Absent genes were treated as missing data. An edge-proportional partition model with proportional branch lengths (-spp) was used, to allow different rate parameters for each partition to accommodate different evolutionary rates between partitions. Nodal support was estimated using ultrafast bootstrap (*n* = 1 000 000) and the 50% consensus tree was reported. For Bayesian analysis, alignments were concatenated to produce a matrix with 1831 positions.

## Results

### Mitochondrial gene structure

**Fig. 1.**
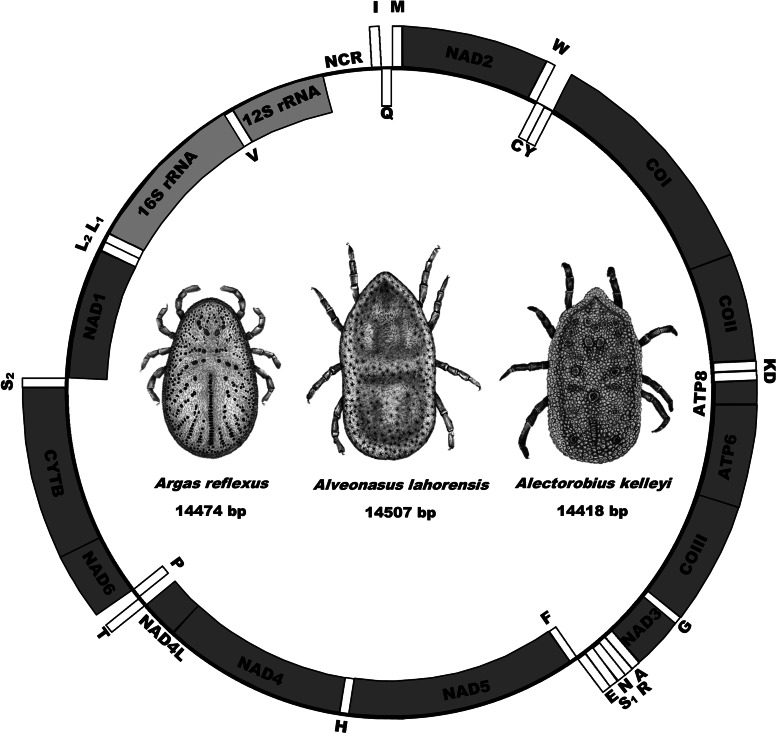
Mitochondrial genome arrangement for *Alveonasus lahorensis*, *Argas reflexus* and *Alectorobius kelleyi*. Genes on the outside are on the forward strand whereas genes on the inside are on the reverse or complementary strand. Protein-encoding genes are in dark grey, ribosomal genes in light grey whereas transfer RNA genes are in white boxes. Also indicated are mitochondrial genome sizes.

The mitochondrial genomes of *A. lahorensis* (PP072240-coverage 273; PP072241-coverage 784), *A. reflexus* (PP072242 – coverage 832) and *A. kelleyi* (SRR23908069 – coverage 167) all possess the standard mitochondrial gene structure and number of genes observed in argasid species (Shao *et al*., 2004; Mans *et al*., 2012;, 2019, 2021; Burger *et al*., 2014; Kneubehl *et al*., 2022) that include the 16S and 12S ribosomal RNA genes, the 13 protein coding genes and the 22 tRNA genes (Fig. 1).

### 16S Ribosomal gene analysis

**Fig. 2.**
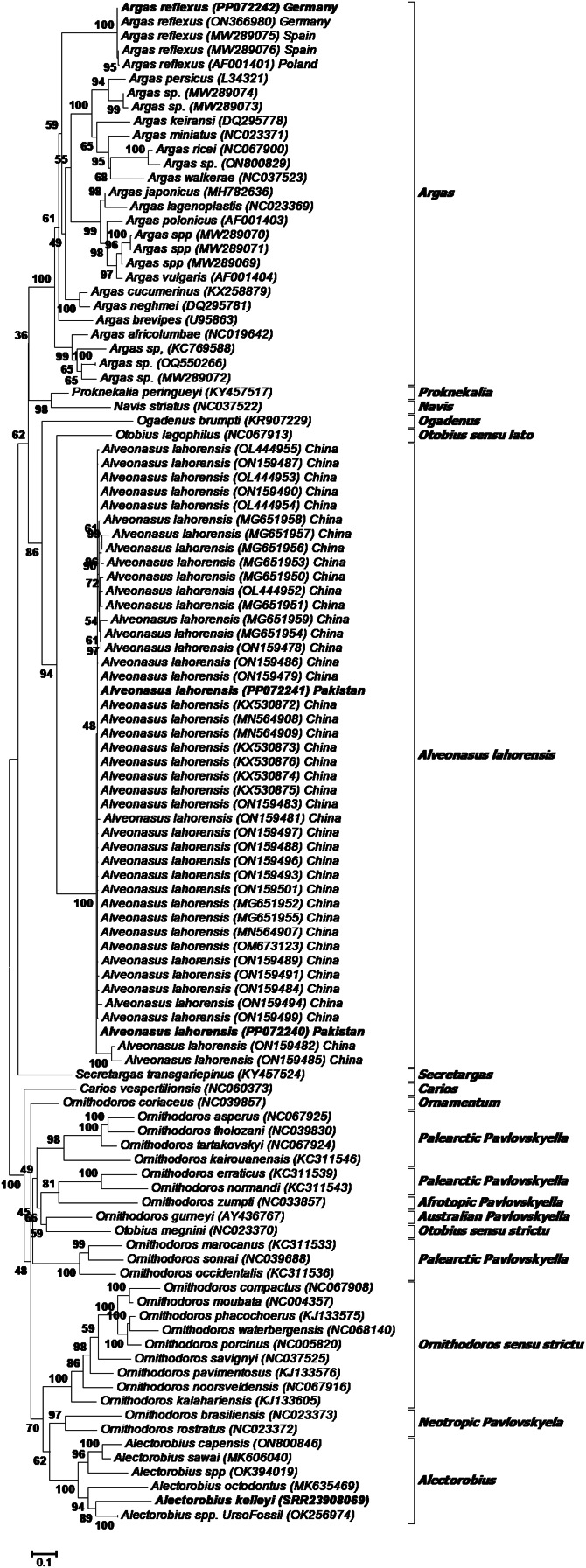
Phylogenetic analysis of the 16S rRNA gene. Indicated are selected members of various genera and subgenera as well as those sequences available in GenBank for *Argas reflexus*, *Alveonasus lahorensis* and *Alectorobius kelleyi*. The current specimens are indicated in bold and GenBank accession numbers in brackets. Bootstrap values are indicated.

A large number of 16S rRNA sequences were previously deposited in GenBank for *A. lahorensis*, but none from the type locality (Pakistan). To confirm the relationship of *A. lahorensis* from Pakistan to published sequences, a BLASTN analysis was performed to determine all closely related sequences, followed by a multiple sequence alignment of all sequences annotated as *A. lahorensis*. All sequences in GenBank (*n* = 41) derive from the Tarim basin in China (Zhao *et al*., 2018), and showed 96.15–99.78% (median 99.35%) sequence identity over a 461 bp region with those from Pakistan. For *A. kelleyi* no 16S rRNA data has yet been deposited in GenBank, while *A. reflexus* shows 99–100% sequence identity to 4 sequences in Genbank that derive from Germany (ON366980), Spain (MW289075, MW289076) and Poland (AF001401). Phylogenetic analysis showed that *A. lahorensis* grouped within a monophyletic clade within the Argasinae (Fig. 2). Within this clade *A. lahorensis* from Pakistan grouped within other sequences from China. *Argas refelxus* grouped within the genus *Argas* with a number of sequences annotated as *A. reflexus*, while *A. kelleyi* grouped within the *Alectorobius* genus (Fig. 2).

### Cytochrome oxidase I gene analysis

**Fig. 3.**
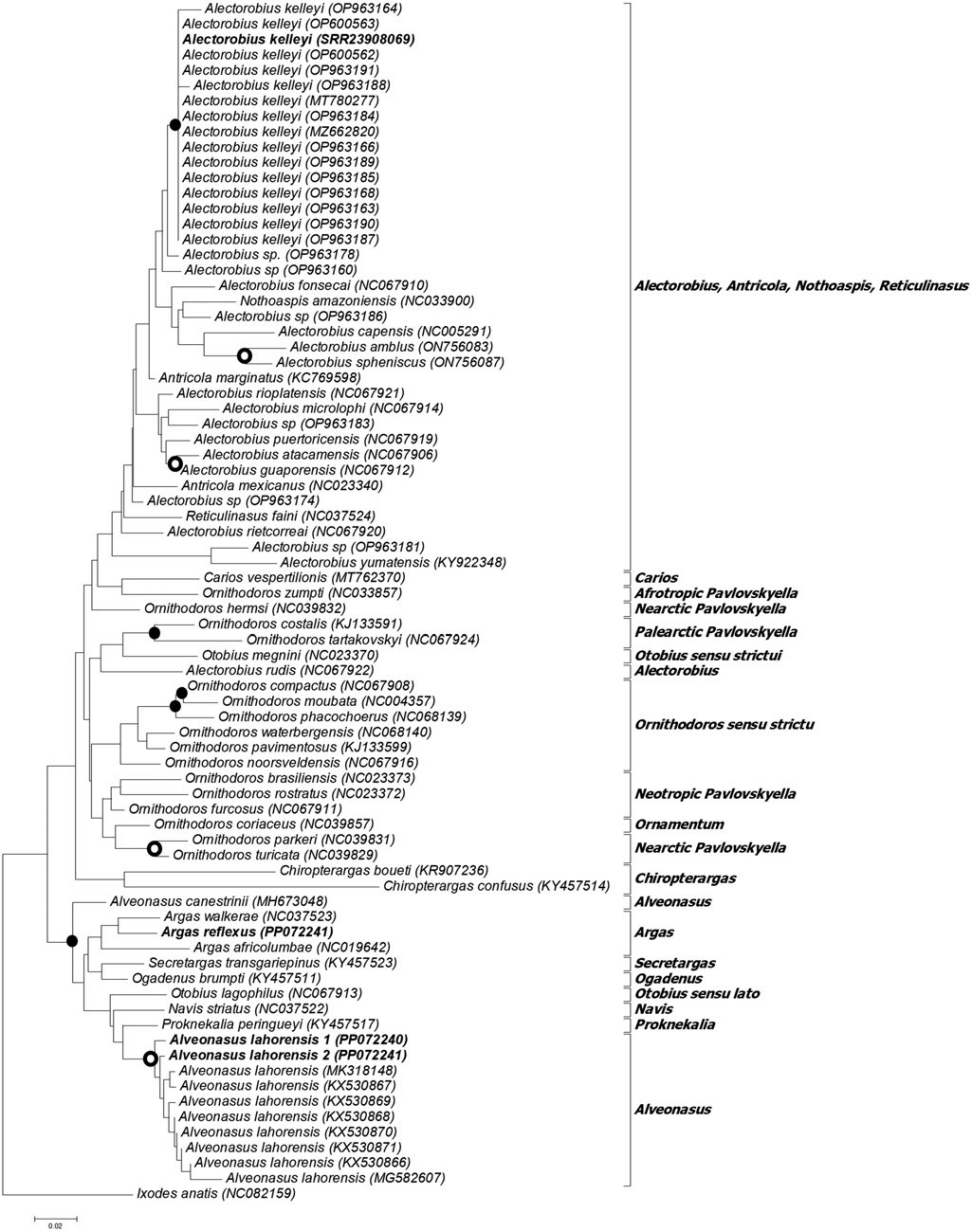
Phylogenetic analysis of the cytochrome oxidase I gene. Indicated are selected members of various genera and subgenera as well as those sequences available in GenBank for *Argas reflexus*, *Alveonasus lahorensis* and *Alectorobius kelleyi*. The current specimens are indicated in bold and GenBank accession numbers in brackets. The tree was rooted using *Ixodes anatis*. Bootstrap values above 70% are indicated with black dots and above 90% with white dots.

Similar to the 16S rRNA gene, a number of *A. lahorensis* COI genes have been deposited in GenBank. A BLASTN analysis was performed to determine all closely related sequences, followed by a multiple sequence alignment of all sequences annotated as *A. lahorensis*. The sequences in GenBank (*n* = 8) derive from the Tarim basin in China (unpublished) and Iran (Hosseini-Chegeni *et al*., 2019), and showed 99.08–99.54% sequence identity over a ~650 bp region. There are no COI genes available for *A. reflexus*, while *A. kelleyi* retrieved 15 hits annotated as *A. kelleyi*, all from the USA, with sequence identities that ranged from 96.20% to 100%. The COI gene for *A. canestrinii* is also available in GenBank (MH673048) and the hypothesis that *Alveonasus* is a monophyletic lineage was tested by performing a phylogenetic analysis of the Argasidae (Fig. 3). All *A. lahorensis* sequences grouped within a well-supported monophyletic clade within the Argasinae. The tree also included *A. canestrinii*, but this sequence did not group in a monophyletic clade with *A. lahorensis*, also previously observed (Hosseini-Chegeni *et al*., 2019), suggesting that *Alveonasus* may be paraphyletic. *Alectorobius kelleyi* grouped within *Alectorobius* in a well-supported clade with other *A. kelleyi* sequences, while *A. reflexus* grouped in a clade with other *Argas* spp.

### Mitochondrial genome analysis

**Fig. 4.**
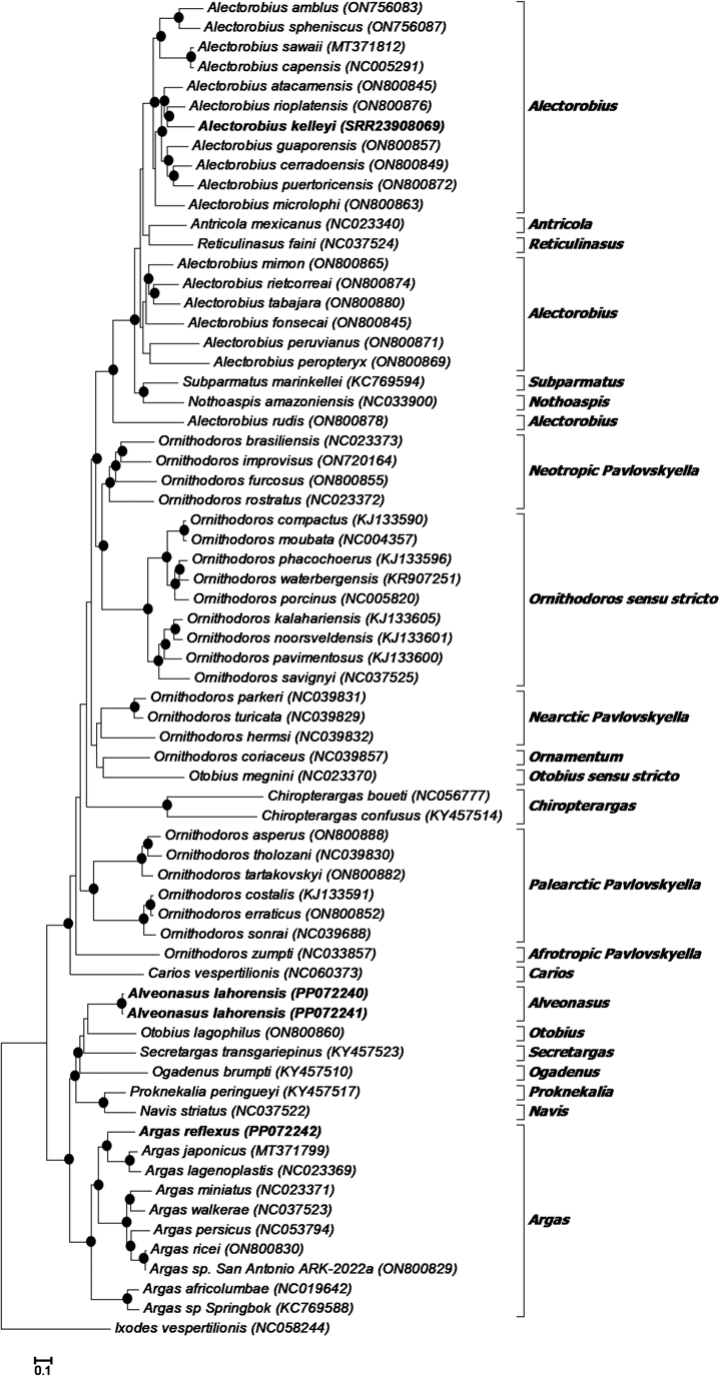
Phylogenetic analysis of the 13 mitochondrial protein coding genes. Indicated are species with their mitochondrial genome accession numbers. The current specimens are indicated in bold. Also indicated are the genera or subgenera of various clades. The tree was rooted using *Ixodes vespertilionis*. Bootstrap values above 90% are indicated with black dots.

Phylogenetic analysis using the 13 protein coding genes indicates that *A. lahorensis* group with good support within the Argasinae in a clade formed by *Alveonasus*, *Navis*, *Ogadenus*, *Otobius lagophilus*, *Proknekalia* and *Secretargas* (Fig. 4). *Argas reflexus* group within the *Argas* clade, while *A. kelleyi* group within the *Alectorobius* clade.

### 18S-28S rRNA gene analysis

**Fig. 5.**
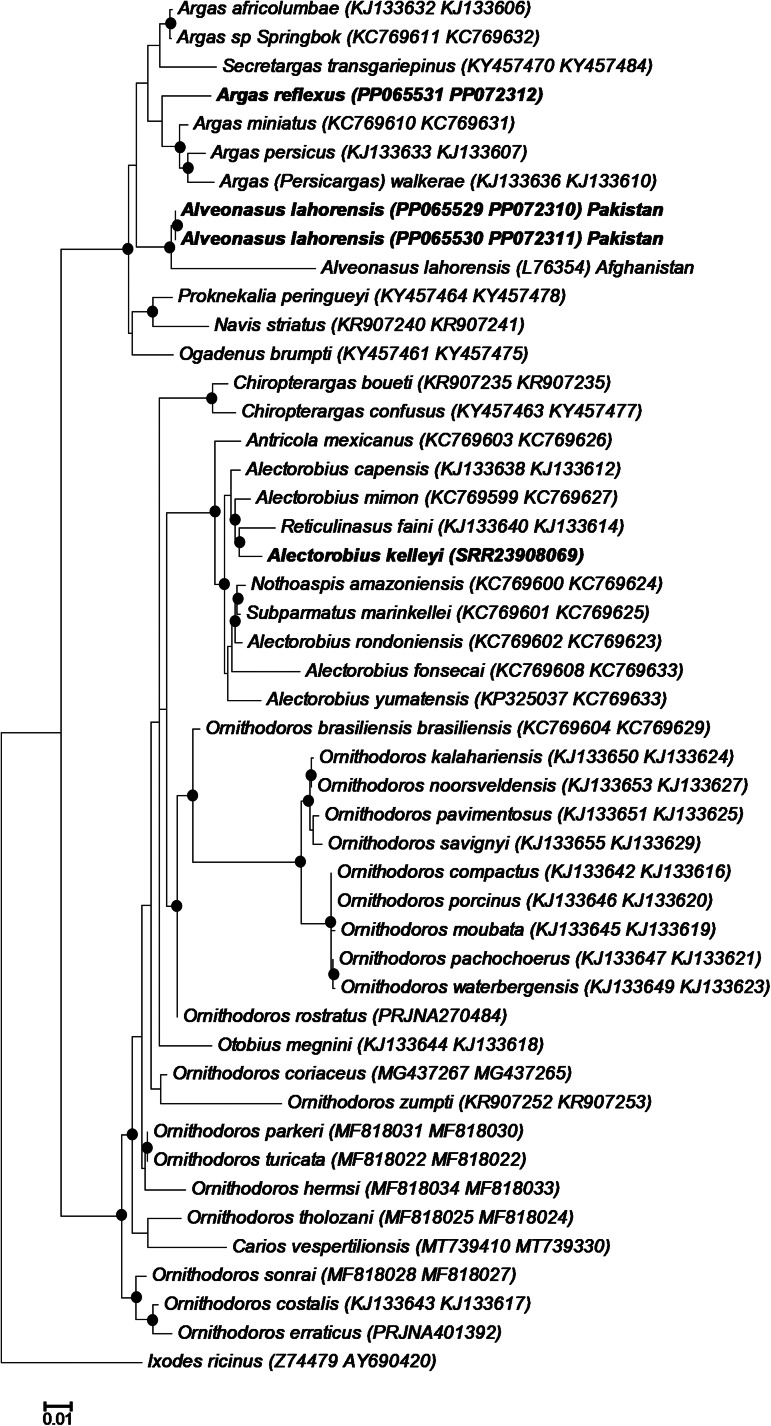
Phylogenetic analysis of the 18S-28S ribosomal RNA genes. Indicated are species with their 18S and 28S rRNA accession numbers in brackets. The current specimens are indicated in bold. The tree was rooted using *Ixodes ricinus*. Bootstrap values above 90% are indicated with black dots.

To confirm that the sequences group within the various subfamilies as indicated for mitogenome analysis, an analysis of the nuclear 18S and 28S ribosomal RNA genes was also performed (Fig. 5). *Alveonasus lahorensis* grouped in the Argasinae with good support. The sequences from Pakistan grouped as sister group to an *A. lahorensis* sequence from Afghanistan (L76354). The sequences from Pakistan showed 100% sequence identity, however, pairwise comparison showed only 95% sequence identity to *A. lahorensis* (Afghanistan). This translates to 42 differences that include 4 gapped positions and would suggest different species (Mans *et al*., 2015). Given the wide geographic distribution of this species, the possibility exist that it is composed of a species complex. Wider geographic sampling is needed to investigate this possibility. *Argas reflexus* grouped within a clade with other *Argas* members, while *A. kelleyi* grouped within a clade formed by members of the *Alectorobius*, *Antricola*, *Reticulinasus* and *Subparmatus* genera.

## Discussion

### Relationship of Alveonasus to other argasid genera

*Alveonasus* was considered a connecting intermediate lineage between *Argas* and *Ornithodoros* (Clifford *et al*., 1964). The latter study included *Proknekalia peringueyi* (Bedford and Hewitt, 1925) and *Proknekalia peusi* (Schulze, 1943) in *Alveonasus* based on their wrinkled integument and absence of the preanal and tranverse anal groove in adults and a large dorsal plate and respiratory apparatus in larvae. *Proknekalia* was raised to its own genus (Keirans *et al*., 1977) as supported by the placement of *Proknekalia* in its own clade in the Argasinae by mitochondrial and 18S-28S rRNA analysis (Mans *et al*., 2019; Mans *et al*., 2021; Current study). Both *Alveonasus* and *Proknekalia* were considered special branches within the genus *Ornithodoros* and attempts to relate these lineages to *Argas* was considered to be based on biological artefacts (Keirans *et al*., 1977). However, the current study shows that both mitochondrial and nuclear genetic data support an association within the Argasinae. Together with the cladistic data from Klompen and Oliver (1993), the evidence that these genera belong within the Argasinae is convincing. It is of interest that *Alveonasus* clusters within a clade formed by *Ogadenus*, *Otobius lagophilus*, *Navis*, *Proknekalia* and *Secretargas*. Hoogstraal (1985) placed *Alveonasus* within the Ornithodorinae, but did recognize a resemblance between *Alveonasus*, *Ogadenus* and *Secretargas*. Pospelova-Shtrom (1969) also placed *Alveonasus* (including *Proknekalia* and *Ogadenus*) and *Otobius* in the Otobiini tribe, although this tribe was placed within the Ornithodorinae. There was therefore recognition of relationships between these genera, perhaps obscured by the conviction that *Alveonasus* had to belong to the Ornithodorinae (Keirans *et al*., 1977). Cladistic analysis placed *Alveonasus*, *Ogadenus*, *Proknekalia* and *Secretargas* into a larger clade of related lineages termed the *Alveonasus* group (Klompen, 1992).

Given that *O. lagophilus* also group within the Argasinae, it is evident that there is an evolutionary relationship with other Argasinae genera. Conversely, the surprising finding that *O. megnini* group within the Ornithodorinae needs to be addressed. The placement of *O. lagophilus* within *Otobius* seemed fairly straightforward. Adults from both species presents a panduriform shape and adults do not feed (Clifford *et al*., 1964; Herring and Beck, 1965). Nymphs present posterodorsal spines, although these differ in number and size between *O. megnini* and *O. lagophilus*, with the latter's spines more slender (Herring and Beck, 1965). Conversely, larvae from *O. lagophilus* lack eyes while *O. megnini* possess 2 pairs of eyes (Herring and Beck, 1965). *Otobius lagophilus* parasitize rabbits exclusively and is found on the face, while *O. megnini* parasitizes a range of domestic animals and is usually found in the ears. *Otobius lagophilus* also completes 1 larval and 1 nymphal moult, while *O. megnini* completes 2 nymphal moults (Herring and Beck, 1965). There is therefore significant differences that may have indicated that these belong to different genera. Even so, both species lack Pd1 palpal setae, a characteristic used to differentiate Argasinae and Ornithodorinae, the former possessing palpal setae Pd1 (Klompen, 1992; Klompen and Oliver, 1993). In the case of *O. lagophilus* these setae would have been lost. Loss of morphological characters within lineages may be more probable than gain of a character *via* independent convergent evolution (homoplasy). Some Argasinae may therefore lack Pd1 palpal setae and this character may not be stable for all members, especially since its functional significance is not apparent for the lineage.

### Paraphyly of the genus *Alveonasus*: larger implications

It may be expected that members from the same genus and geographic region should share a recent common ancestor and therefore group as sister clades. However, even though *A. canestrinii* and *A. lahorensis* occur in the same region (Hosseini-Chegeni *et al*., 2019), they do not group as sister clades or even as a monophyletic clade to the exclusion of other genera (Fig. 3). Cladistic analysis of larval characters also failed to provide support for a monophyletic *Alveonasus* when *A. canestrinii*, *A. eboris* and *A. lahorensis* was compared (Klompen, 1992). Additional support for paraphyly may be found in the unique biology of *A. lahorensis* which is the only argasid species to display 2-host behaviour with the larvae and nymphs feeding on the same host for up to 3–6 weeks before the engorged third-instar nymph drops and moults to an adult (Hoogstraal, 1985). This suggests that *Alveonasus* may be comprised of different independent lineages (i.e. genera). In fact, given the wide geographic distribution observed for members of *Alveonasus*, it may be expected that *Alveonasus* may likely be comprised of a large number of independent lineages, probably linked to their diverse geographic distribution (Table 1). Indeed, a similar phenomenon is seen for the subgenus *Pavlovskyella*, where different geographic lineages group as unique clades in the Ornithodorinae (Mans *et al*., 2019). It also implies that the eponymous feature of *Alveonasus*, the madreporian sculpturing of the integument (Clifford *et al*., 1964), evolved independently or derive from similar, but independent developmental pathways (Mans, 2023). If an ancestral developmental pathway for the *Alveonasus* sensu Klompen (1992) group was present, perhaps characterized by polygonal depressions surrounded by ridges, formed by integumental folds from the centre of these depressions (Klompen and Oliver, 1993), it resulted in numerous integumental variations, some madreporean and others more accentuated by the ridges. Depending on how the integumental development plan unfolds in each species, the specific patterns observed may not be stable enough for genus level classification.

Given the recent surprising placement of *O. lagophilus* in the Argasinae (Kneubehl *et al*., 2022), the placement of other members of *Alveonasus* in the Argasinae (and in *Alveonasus*) should be empirically confirmed. While it is likely that all members would eventually group within a larger clade formed by *Alveonasus*, *Navis*, *Ogadenus*, *Proknekalia*, *Secretargas* and *O. lagophilus* (the *Alveonasus* group sensu Klompen, 1992), this cannot be taken for granted. A concerted effort to place the remaining *Alveonasus* species will be important, since it is likely that the unexpected grouping of the remaining lineages may profoundly impact our understanding of the evolution of this group.

## Conclusion

The current study showed that the type species for *Alveonasus* group within the Argasinae and not the Ornithodorinae as suggested by the American (Clifford *et al*., 1964; Hoogstraal, 1985; Guglielmone *et al*., 2010), Russian (Pospelova-Shtrom, 1946, 1969) and French (Camicas and Morel, 1977; Camicas *et al*., 1998) schools. Inclusion of *Alveonasus* in the Argasinae has been indicated by previous molecular studies using nuclear 18S rRNA and mitochondrial 16S rRNA (Black *et al*., 1997; Zhao *et al*., 2018). However, the inclusion of *Alveonasus* in the Argasinae was not specifically discussed nor proposed in these studies, nor recognized by the tick community (except for Mans *et al*., 2019 and Mans *et al*., 2021). The current study is therefore the first to provide evidence that unambiguously places *Alveonasus* in the Argasinae and recognize its placement in this subfamily, after the proposal by Klompen and Oliver (1993), that *Alveonasus* should be placed in the Argasinae based on cladistic analysis. This is also one of the last major genera for which mitochondrial genome data has been reported. The only formally recognized genus or lineage for which mitochondrial genome data has not yet been reported is the subgenus *Microargas* (monotypic species *Ornithodoros transversus* Banks (1902)). It is becoming clear that many more evolutionary independent lineages may exist in the Argasidae that may warrant generic status that include members from *Alectorobius*, *Pavlovskyella*, *Otobius* and now *Alveonasus* (Mans, 2023).

## Data Availability

All data in the study is available in the public databases as listed by their accession numbers.
