# Mitochondrial genome and nuclear ribosomal RNA analysis place *Alveonasus lahorensis* within the Argasinae and suggest that the genus *Alveonasus* is paraphyletic – ERRATUM

**DOI:** 10.1017/S0031182024001537

**Published:** 2024-08

**Authors:** Ben J. Mans, Lidia Chitimia-Dobler, Ronel Pienaar, Minique de Castro, Mehran Khan, Mashal M. Almutairi, Abdulaziz Alouffi, Abid Ali

**Affiliations:** 1 Epidemiology, Parasites and Vectors, Agricultural Research Council-Onderstepoort Veterinary Research, Onderstepoort 0110, South Africa; 2 Department of Life and Consumer Sciences, University of South Africa, South Africa; 3 Department of Zoology and Entomology, University of the Free State, Bloemfontein 9301, South Africa; 4 Bundeswehr Institute of Microbiology, Munich, Germany; 5 Fraunhofer Institute of Immunology, Infection and Pandemic Research, Penzberg, Germany; 6 The Biotechnology Platform, Agricultural Research Council-Biotechnology Platform, Onderstepoort 0110, South Africa; 7 Department of Zoology, Abdul Wali Khan University Mardan, Mardan 23200, Pakistan; 8 Department of Pharmacology and Toxicology, College of Pharmacy, King Saud University, Riyadh 11451, Saudi Arabia; 9 King Abdulaziz City for Science and Technology, Riyadh 12354, Saudi Arabia

We apologise for an error in this paper, whereby the incorrect Acknowledgements section was published. The correct acknowledgement section is as follows:
